# The Nature of Rehabilitation Programs to Improve Musculoskeletal, Biomechanical, Functional, and Patient-Reported Outcomes in Athletes With ACL Reconstruction: A Scoping Review

**DOI:** 10.1177/19417381231158327

**Published:** 2023-03-17

**Authors:** Kelly Poretti, Navid Ghoddosi, Joel Martin, Oladipo Eddo, Nelson Cortes, Nicholas C. Clark

**Affiliations:** † School of Kinesiology, George Mason University, Manassas, Virginia; ‡ Sports Medicine Assessment Research and Testing (SMART) Laboratory, George Mason University, Virginia; § School of Sport, Rehabilitation, and Exercise Sciences, University of Essex, Colchester, Essex, UK; ‖ Department of Bioengineering, George Mason University, Fairfax, Virginia

**Keywords:** knee, lower extremity, sports medicine

## Abstract

**Context:**

After anterior cruciate ligament (ACL) reconstruction (ACLR), athletes commonly undergo prolonged rehabilitation (eg, 9-12 months), but few actually return to preinjury sports activities. The nature (composition, configuration) of an ACL rehabilitation program (ACL-RP) is an important factor in determining rehabilitation outcomes; however, details about the nature of ACL-RPs are reported inconsistently in research studies. To guide future research reporting to support clinical translation and implementation of ACL-RPs, it is necessary to describe the nature, reporting, and outcomes of ACL-RPs in the current literature.

**Objective:**

The purpose of this scoping review was to understand the nature and reporting of various ACL-RPs that address musculoskeletal, biomechanical, functional, or patient-reported outcome measures in adult and pediatric athletes with ACLR.

**Data Sources:**

Articles were selected from searches in 5 electronic databases (PubMed, EbscoHost [MEDLINE, SportDiscus, CINAHL Plus], PROQuest, Cochrane, and Embase).

**Study Selection:**

Studies were included if they evaluated a post-ACL-RP that implemented strength, balance, plyometric, change of direction running, and/or agility running and included self-reported physical function, quality of life, or pain outcomes.

**Study Design:**

Scoping review using the Preferred Reporting Items for Systematic Reviews and Meta-Analyses extension for scoping reviews (PRISMA-ScR) guidelines.

**Level of Evidence:**

Level 4.

**Data Extraction:**

Data were extracted and synthesized to evaluate the reporting of acute program variables (APVs) and exercise descriptors (EDs); 17 studies were included in the final synthesis.

**Results:**

Studies reported between 0% and 67% of the APVs and EDs combined. Only 2 studies were considered to have adequate reporting of both APVs and EDs.

**Conclusion:**

Inadequate reporting of APVs and EDs in past studies restricts the translation and implementation of existing research-based ACL-RPs to present-day clinical contexts.

Over 175,000 anterior cruciate ligament (ACL) reconstructions (ACLRs) are performed each year in the United States alone to restore knee mechanical joint stability for athletes to resume activities.^6^ However, outcomes after ACLR continue to be poor, with only one-third of athletes returning to preinjury sports activities.^9^ Aside from time loss from sport with ACLR, many athletes commonly experience persistent knee impairments,^30^ physical activity limitations,^30^ reduced quality of life,^17^ and early-onset knee osteoarthritis.^17^ After ACLR, rehabilitation takes place with training that commonly includes a variety of exercises performed in a systematic progression to return the athlete to sport.^4,12^ Common classifications of rehabilitation exercises include strength,^12^ balance,^12^ plyometric,^12^ change of direction running,^34^ and agility running.^34^ A growing body of literature reports the outcomes of the return-to-sport phase of rehabilitation after ACLR.^1,2,6,45^

The “return-to-sport” phase of rehabilitation occurs toward the end of the rehabilitation process,^9^ which consists of general categories and progressions of “high-impact” and “high-risk” exercises (eg, jumping, hopping).^34^ This phase prepares the athlete for a successful return-to-sport activities.^34^ Exercise interventions employed in the return-to-sport phase are typically classified as: strength,^12^ balance,^1,12^ plyometric,^12^ change of direction running,^34^ and agility running.^34^ Previous literature has explored the effectiveness of ACL rehabilitation programs (ACL-RPs) incorporating unimodal exercise intervention (ie, strength training alone)^9,18,24,28,33,43,44^; however, there is limited literature evaluating multimodal ACL-RPs (ie, strength, plyometric, and agility exercises) and/or comparing outcomes with unimodal RPs. Furthermore, adequate reporting of acute program variables (APVs; eg, exercise order, number of sets, number of repetitions, intensity, between-set duration, weekly frequency of session, number of rest days between sessions) and exercise descriptors (EDs; eg, single- or double-leg exercise, loading method, total duration of the program, supervision, progression) are needed for safe and effective translation of research-based RPs to real-world clinical practice.^23,28,35^ To this end, guidelines have been developed to promote adequate reporting of therapeutic exercise programs.^35,39^

The purpose of this scoping review was to understand the nature and reporting of various RPs that address musculoskeletal, biomechanical, functional, or patient-reported outcome measures in athletes with ACLR. We use the term “nature” to refer to the classification and composition (APVs) of the RP, and the configuration of its exercises (EDs). The specific aim was to describe the reporting of APVs and EDs for strength, balance, plyometric, change of direction running, agility running exercise, and other/metabolic conditioning components in existing published research with athletes with ACLR. The desired outcomes of this scoping review were to (1) assess existing ACL-RPs for adequate reporting that support the clinician’s ability to replicate and translate research-based RPs to real-world clinical practice and (2) provide insight into the utility of comprehensive reporting of APVs and EDs to facilitate translation to clinical practice.

## Methods

### Protocol and Registration

The review was conducted and reported according to the Preferred Reporting Items for Systematic Reviews and Meta-Analyses extension for scoping reviews (PRISMA-ScR) guidelines.^41^ A review protocol was published on the Open Science Framework in December 2021.^32^

### Eligibility Criteria

The following inclusion criteria were used to determine study eligibility: adult and pediatric athletes; published from 1990 to 2021 only (because ACLR surgical techniques shifted from arthrotomy to arthroscopy in the 1990s)^14,45^; ACLR studies with/without concomitant medial collateral ligament injury; ACLR studies with/without concomitant meniscal injury; included a postsurgery RP and a comparison condition; the intervention included strength, balance, plyometric, change of direction running, or agility running exercises; reported on musculoskeletal, biomechanical, functional, and/or patient-reported outcomes; study designs were randomized controlled trials (RCTs), quasi-RCTs, nonrandomized controlled trials, or uncontrolled trials; and studies were peer-reviewed and published in English. Studies excluded were those with a population that did not have ACLR, nonintervention studies, and unpublished studies.

### Information Sources and Search Terms

A search was conducted that utilized PubMed, EbscoHost (MEDLINE, SportDiscus, CINAHL Plus), PROQuest, Cochrane, and Embase. The search terms listed in Appendix Table A1 (available online) were applied to each database: the search was conducted in January 2022.

### Selection of Sources of Evidence

Title/abstract screening was undertaken by 2 authors. Full texts were reviewed independently by 2 authors, and conflicts were addressed by a 3rd author as needed. Eligibility was determined by the criteria stated previously. Pilot testing was completed to consistency among the authors screening the articles.

### Data Charting Process

After identifying potential manuscripts for review, SciWheel (https://www.sciwheel.com) was utilized to organize, store, and deduplicate results. Data extraction and charting were undertaken by 2 authors separately using a specifically designed synthesis matrix. Extraction and charting were then reviewed by the remaining 4 authors to reach a consensus. Data were obtained directly from the published articles only.

Extracted data were recorded in an Excel file developed specifically for this review. The Excel file consists of 2 tables: one for general information about the manuscripts and the other to allow for more detailed information about the exercise program in the selected manuscripts.

### Data Items

The following data were extracted: study design; participant mean age, sex, and sport; sample size; athlete level; ACL surgery characteristics and time since surgery; graft type; primary musculoskeletal, biomechanical, functional, and patient-reported outcomes; overall findings; and rehabilitation/intervention characteristics (length of intervention, types of exercises incorporated, variables reported). Definitions of exercise intervention terms and sports level terms used were agreed upon by all authors to eliminate any potential discrepancies between articles (Appendix Table A2, available online).^8,10,11,13,16,19,26,46,47^ The study programs were then analyzed to determine whether each of the APVs and EDs listed in Appendix Table A3 (available online) were reported for each of the exercises in each program.

Post ACL-R rehabilitation programs generally vary in terms of exercise types and the implementation of those exercises, which are typically reported through the APV and EDs.^15,28,34,35^ Since replication of ACL-R rehabilitation programs is dependent on the adequate reporting of APVs and EDs regardless of program effectiveness, we focused on the frequency of APV/ED reporting and the types of programs with high frequencies similar to previous work.^3,23^ The data from the literature was organized thematically according to the exercise and program types, APVs, and EDs.

### Quality Appraisal of Individual Sources of Evidence

This scoping review primarily examined the reporting of APVs and EDs and did not analyze the outcomes or effectiveness of any interventions. Therefore, study quality, including the risk of bias, was not relevant to this review, and a quality appraisal of the selected articles was not included.

### Synthesis of Results

Overall reporting frequencies for APVs and EDs were calculated based on the total number of exercises from all studies included in this scoping review. The frequency was equal to the number of reported APVs or EDs divided by the total number of those values that could be reported. These values were then calculated based on each study and the exercise type separately. For this scoping review, “adequate” reporting of APVs and/or EDs was defined as ≥51%, to represent the majority, as used in previous literature.^23^

## Results

### Selection of Sources of Evidence

**Figure 1.**
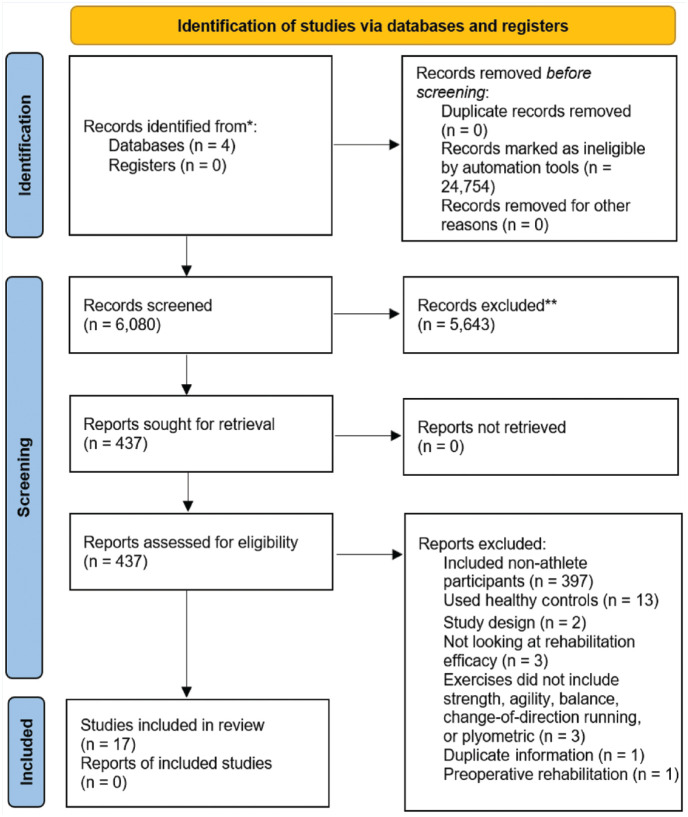
PRISMA flow diagram: identification of studies. PRISMA, Preferred Reporting Items for Systematic Reviews and Meta-Analyses.

In all, 6080 titles and abstracts were screened (Figure 1). A total of 437 full-text reports were then screened for eligibility and 17 studies were identified as meeting the inclusion/exclusion criteria for this scoping review. The most common reasons for exclusion were studies that included nonathlete participants or used healthy controls.

### Characteristics of Sources of Evidence

All studies were published in either 2002 or after (Appendix Figure A1, available online). Among the 17 included studies, 11 were RCTs,^1,2,5-7,21,27,29,31,36,43^ and 6 were longitudinal controlled studies (Appendix Table A4, available online).^18,20,22,38,40,42^ These studies included 11 with adult populations,^18,20-22,27,29,31,36,40,42,43^ and 6 with pediatric and adult populations.^1,2,5-7,38^ Seven studies included only male participants,^2,5,6,36,40,42,43^ 1 study included only female participants,^7^ and 9 studies included both male and female participants.^1,18,20-22,27,29,31,38^ Data extracted from the included studies in this scoping review can be found in the Appendix (available online).

## Synthesis of Results

Overall, studies reported between 0% and 67% of the APVs and EDs combined (Appendix Figure A2, available online). Only 2 studies were considered to have adequate reporting of both APVs and EDs.^18,43^ Between both the APVs and EDs, number of repetitions was reported the most, at a frequency of 51% across all studies, and exercise order was reported the least, at a frequency of 0% across all studies (Appendix Figure A3, available online). Overall, APV and ED reporting was inadequate, as only 1 APV was adequately reported out of the total of 7 analyzed. None of the EDs were adequately reported (Appendix Figure A3, available online).

### APV Reporting

APVs were reported as between 0% and 51% for all studies (Appendix Figure A4, available online). No included studies reported exercise order. However, the number of repetitions was reported adequately across all included studies, with a frequency of 51%. When broken down by exercise type (Appendix Figure A5, available online), none of the exercise types adequately reported APVs.

### ED Reporting

Reporting of EDs occurred between 4% and 39% (Appendix Figures A2 and A6, available online) and EDs were generally reported at higher rates in comparison with APVs (Appendix Figures A2, A4, and A6, available online). However, none of the EDs were reported adequately across the included studies. In addition, when broken down by exercise type (Appendix Figure A7, available online), none of the exercise types adequately reported EDs.

### Composition of Rehabilitation Programs

Of the 17 total studies included, 5 reported using only strength exercises,^18,20,27,29,43^ 3 reported using strength and metabolic exercises,^21,22,31^ 1 study included strength and agility exercises,^38^ 1 study included strength and balance exercises,^2^ and a total of 7 studies included more than 3 exercise types (Appendix Table A5, available online).^1,5-7,36,40,42^

## Discussion

### Summary of Evidence

The purpose of this scoping review was to understand the nature and reporting of APVs and EDs for strength, balance, plyometric, change of direction running, agility running, or metabolic conditioning/other to understand whether current ACL-RPs adequately report details for clinician replication in practice. In general, the results show that studies do not adequately report APVs and EDs of ACL-RPs in a way that is useful for clinical practice. Only 1 APV, number of repetitions, was reported adequately throughout the included studies.^1,5,7,18,20,40,42,43^ All other APVs and EDs were reported inadequately across the studies included in this review across all exercise types^1,2,5-7,18,20-22,27,29,31,36,38,40,42,43^; this represents a major obstacle for clinicians looking to translate research-based ACL-RPs into their day-to-day clinical practice.^3,23^

RPs incorporating strength,^18,20-22,27,29,43^ balance,^2^ plyometric,^1,5,40^ change of direction running,^40^ and/or agility^40^ exercises have shown positive effects on selected outcomes after ACLR.^3,15,24,34^ However, due to inadequate reporting of APVs and EDs, it is not possible to draw practical and meaningful conclusions regarding how the specific composition and configuration of these exercises/programs may have affected athletes with ACLR.^3^ In addition, lack of reporting of APVs and EDs results in the inability of other researchers to either replicate or build upon findings,^25^ the inability of clinicians to implement programs and in general adopt practices guided by research,^25,39^ and concern for patient safety when implementing these programs.^23,39^ From a safety perspective, APVs and EDs provide the appropriate information needed by clinicians to ensure that they are protecting the injured leg with the appropriate range of motion points during specific timepoints in the rehabilitation process or with the appropriate loading mechanism to best control the rehabilitation at certain timepoints.^23^ Likewise, from an efficacy perspective, these APVs and EDs can be manipulated to target specific weaknesses or can load the injured leg appropriately to elicit the response needed for rehabilitation and safe return to play.^23^

The Consensus on Exercise Reporting Template (CERT) and the Template for Intervention Description and Replication (TIDieR) are 2 templates that are currently available to be used alongside the Consolidated Standards of Reporting Trials (CONSORT) 2010 statement to aid in better reporting in research studies.^25,37,39^ It is essential to improve the accuracy and completeness of research reports to enhance the quality of research and its repeatability.^35^ Future studies should consider utilizing these tools to improve the translation of research-based rehabilitation into clinical practice.

### Study Limitations

Although we limited the date range to control for the evolution of ACLR surgical methods across time, it is possible that eligible studies published before 1990 were excluded, along with studies and publications that were excluded due to the language restrictions applied for the purposes of this scoping review. In addition, while studies involving people with ACL deficiency may offer a more comprehensive picture of ACL rehabilitation, we limited our search to studies that focused exclusively on ACLR. Furthermore, our search criteria excluded studies that utilized healthy participants in the control group.

### Conclusion

The reporting of APVs and EDs is generally inadequate in ACLR rehabilitation studies across strength training, balance training, plyometric training, change of direction running drills, and agility running drills. The inadequate reporting of APVs and EDs restricts the translation of research-based RPs to clinical practice. The significance of this review is that it identifies the need for improved reporting in future studies evaluating the impacts of ACL-RPs on athletes’ rehabilitation outcomes. This will facilitate better interpretation of research findings, translation and adoption of research in clinical settings, and athlete safety.

## Supplemental Material

sj-pdf-1-sph-10.1177_19417381231158327 – Supplemental material for The Nature of Rehabilitation Programs to Improve Musculoskeletal, Biomechanical, Functional, and Patient-Reported Outcomes in Athletes With ACL Reconstruction: A Scoping Review Supplemental material, sj-pdf-1-sph-10.1177_19417381231158327 for The Nature of Rehabilitation Programs to Improve Musculoskeletal, Biomechanical, Functional, and Patient-Reported Outcomes in Athletes With ACL Reconstruction: A Scoping Review by Kelly Poretti, Navid Ghoddosi, Joel Martin, Oladipo Eddo, Nelson Cortes and Nicholas C. Clark in Sports Health: A Multidisciplinary Approach
