# Supplementary material for: The Nature of Rehabilitation Programs to Improve Musculoskeletal, Biomechanical, Functional, and Patient-Reported Outcomes in Athletes With ACL Reconstruction: A Scoping Review
Source: Sports Health. 2023 Mar 17;16(3):390–5. doi: 10.1177/19417381231158327 (PMC11025522; doi:10.1177/19417381231158327)
Supplement: sj-pdf-1-sph-10.1177_19417381231158327 – Supplemental material for The Nature of Rehabilitation Programs to Improve Musculoskeletal, Biomechanical, Functional, and Patient-Reported Outcomes in Athletes With ACL Reconstruction: A Scoping Review [file sj-pdf-1-sph-10.1177_19417381231158327.pdf]

Online Appendix

Table A1: Search Terms – The search was conducted using the items listed in the table below in the following order: (1 AND 2) AND (3 OR 4 OR 5) OR 6.

Item	Desired Outcome	Search Terms
1	ACL reconstruction	ACL reconstruction OR anterior cruciate ligament reconstruction
2	Intervention	Intervention OR rehab* OR exercise OR treatment OR train*
3	Musculoskeletal Outcomes	Quad* strength OR hamstring* strength OR flexor* strength OR extensor* strength OR resistance train*
4	Biomechanical Outcomes	KAM OR knee moment OR knee abduction moment
5	Functional outcomes	Running OR change of direction OR agility OR change-of-direction OR Balance OR plyometric*
6	Patient-reported outcomes	Pain OR quality of life OR QoL OR KOOS

Table A2: Definitions of Key Terms

**Term**	**Definition**
Exercise Interventions	
Muscle Strength	The ability of a muscle to produce force.[11]
Muscle Strength Training	Any form of exercise primarily directed at increasing the ability of a muscle or muscle group to produce force.[13]
Balance	The ability to maintain the body's center of mass and center of pressure within its baseline of support via internal moments countering external moments that act to destabilize the body and its joints.[26]
Balance Training	Any form of exercise primarily directed at increasing the ability to maintain the body's center of-mass and center-of-pressure within its base-of-support.[26]

Plyometrics	Movements that include a rapid stretch-shortening cycle and enable a muscle to generate high levels of force in as short a time as possible.[10]
Plyometric Training	Any form of exercise that includes a rapid stretch-shortening cycle and is primarily directed at increasing the ability of a muscle to generate high levels of force in as short a time as possible.[10]
Change of Direction	The ability to change the velocity and direction of body movement rapidly in pre-planned contexts.[47]
Change of Direction Training	Any form of exercise primarily directed at increasing the ability to change the velocity and direction of body movement rapidly in pre-planned contexts.[47]
Agility	The ability to change the direction or velocity of body movement rapidly in response to an external stimulus in unplanned contexts and without loss of balance.[8]
Agility Training	Any form of exercise primarily directed at increasing the ability to change the direction or velocity of body movement rapidly in response to an external stimulus and without loss of balance.[47]
Metabolic Training	Any form of exercise primarily directed at developing both aerobic and anaerobic energy systems and producing metabolic adaptation in athletes.[19,46]
Sports Level	
Recreational	Individuals with a low level of commitment, little time involvement, and who merely play at the game; dabblers.[16]
Amateur	Individuals are not paid to perform. Individuals are defined by a high level of commitment to excellence coupled with pursuit of activity for pleasure.[16]
Professional	Individuals are paid to perform. Individuals are defined by being the most specialized and highly trained coupled with pursuit of activity for employment.[16]

Table A3: Acute Program Variables and Exercise Descriptors

Acute Program Variables (APVs)	Exercise Descriptors (EDs)
Exercise order	*Single- or double- leg exercise*
Number of sets	*Loading method*
Number of repetitions	*Total duration of the program*
Intensity	*Unsupervised or supervised*
Between-set duration	*Progression*
Weekly frequency of session	
Number of rest days between sessions	

Appendix Table A4: General Study Characteristics

Author(s)	Year of Pub	Study Design	Sample Size	Participant Age	Population: Pediatric, adult	Participant sex	Sport	ACL surgery characteristics	Graft Type (if reported)	Time since ACL reconstruction surgery
Majima	2002	Randomized Control (prospective comparative study)	62	Mean 24.5 range from 18-42	Adult	26 F, 36 M	NA	The semitendinosus and gracilis tendons were harvested using a tendon stripper. Each end of the doubled tendons in a series was connected with polyester tape. The intraarticular portion of the surgery was done using tourniquet control.	Doubled semitendinosus and gracilis tendon autograft	Immediate (accelerated group) or 1 week (conservative group)
Tsaklis	2002	Longitudinal controlled study	45	24.8 ± 5.6 yrs	Adult	All males	football 18 (40%), basketball 12(27%), track and field 5(11%), volleyball 3(7%), Tae Kwon Do 1(2%), cycling 1(2%) and amateur athletes 5–11.1%	Operation was due to ACL lesion, patellar tendon bone graft	Patellar tendon bone graft	1 month post operative rehabilitation then 2 months of rehabilitation under this protocol (at the end of the study the participants were 3 months post-op
Liu-Ambrose	2003	Randomized Clinical Control	10	ST = 24.7 (2.7), PT = 25 (3.7)	Adult	ST = 3 M, 2 F PT = 1 M, 4 F	NA	NA	Standardized ipsilateral semitendinosus tendon ACL reconstruction	ST = 12.2 (8.3) months, PT = 6.7 (0.8) months
Gerber	2007	Longitudinal controlled study, randomized, matched	40	18-50	Adult	Both	NA	Two surgeons performed all of the ACLR surgeries and used arthroscopically assisted technique. For the semitendinosus-gracilis procedure, a 3.5-cm incision was made over the medial aspect of the tibia, directly over the semitendinosus and gracilis tendons. Sutures were placed in a whip-stitch fashion in the distal 2.5 cm of each of the tendons. There are more detailed information about surgery in this article	Hamstring graft:20, Patellar tendon graft:20	Three weeks after surgery

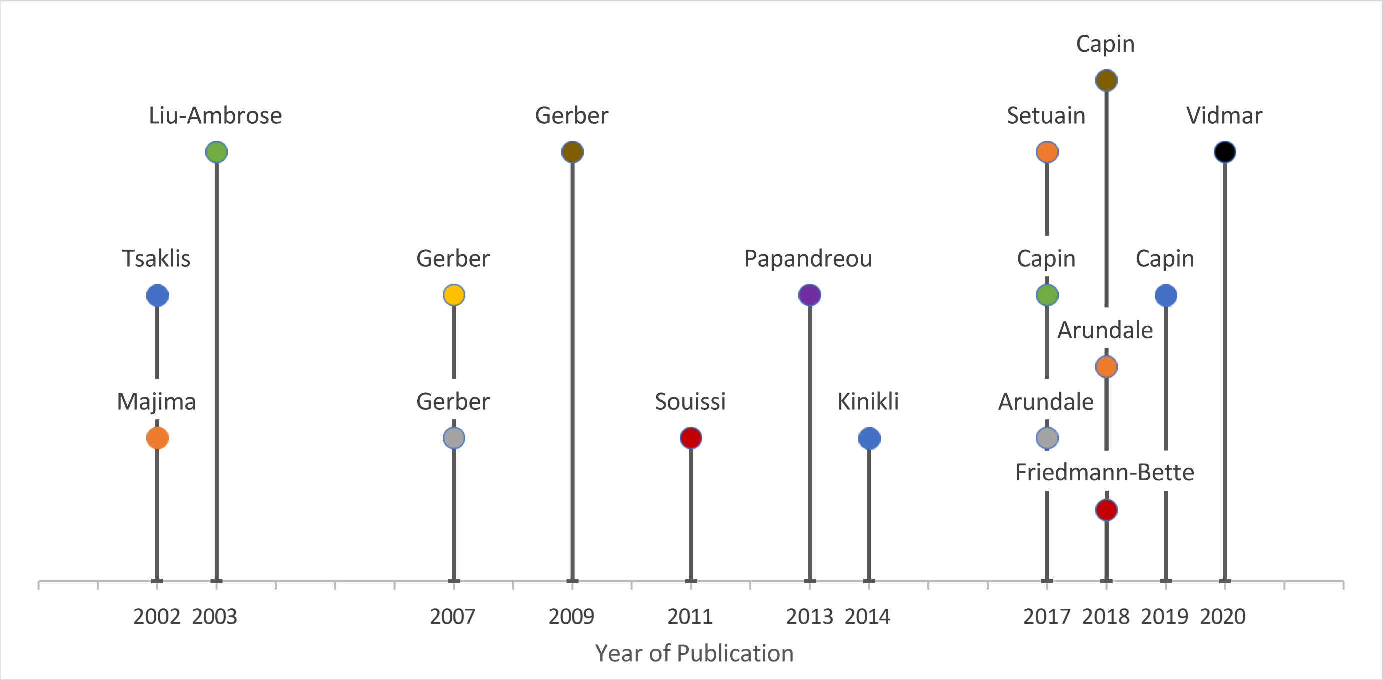

Figure A1: Timeline of the included studies based on publication year.

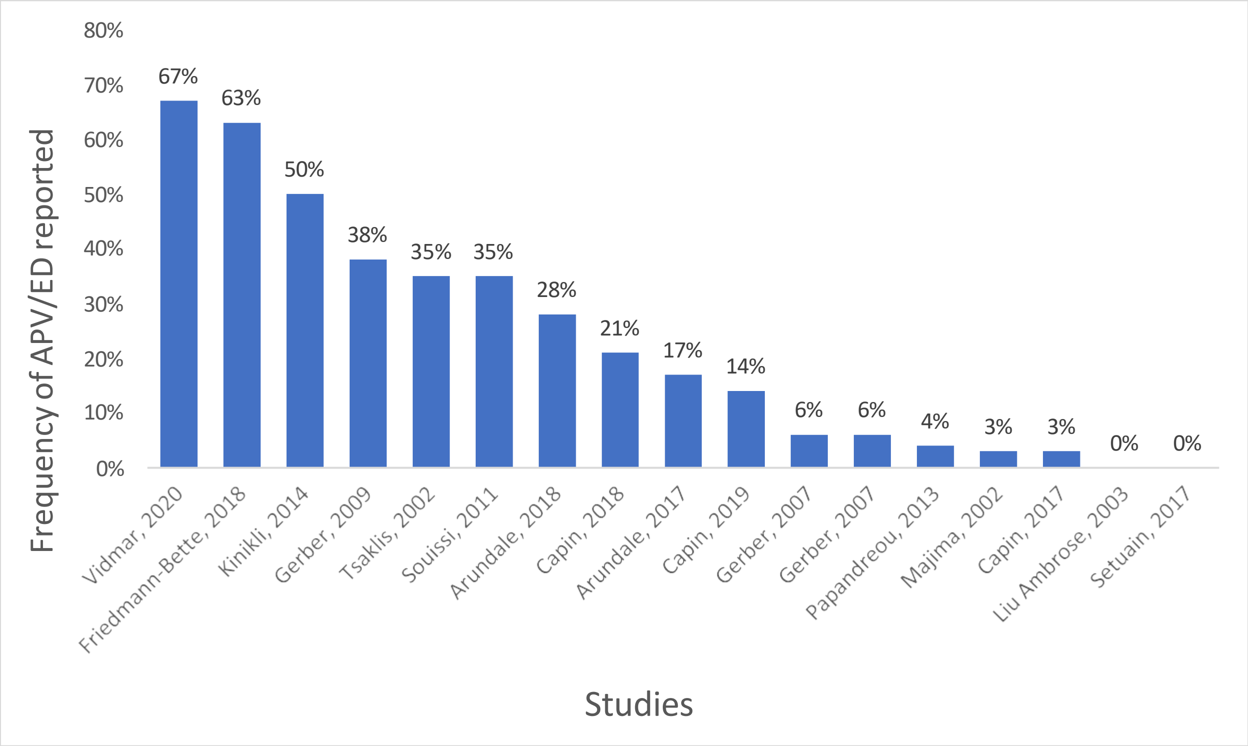

Figure A2: Total Reporting Frequencies of APVs and EDs Combined Per Study

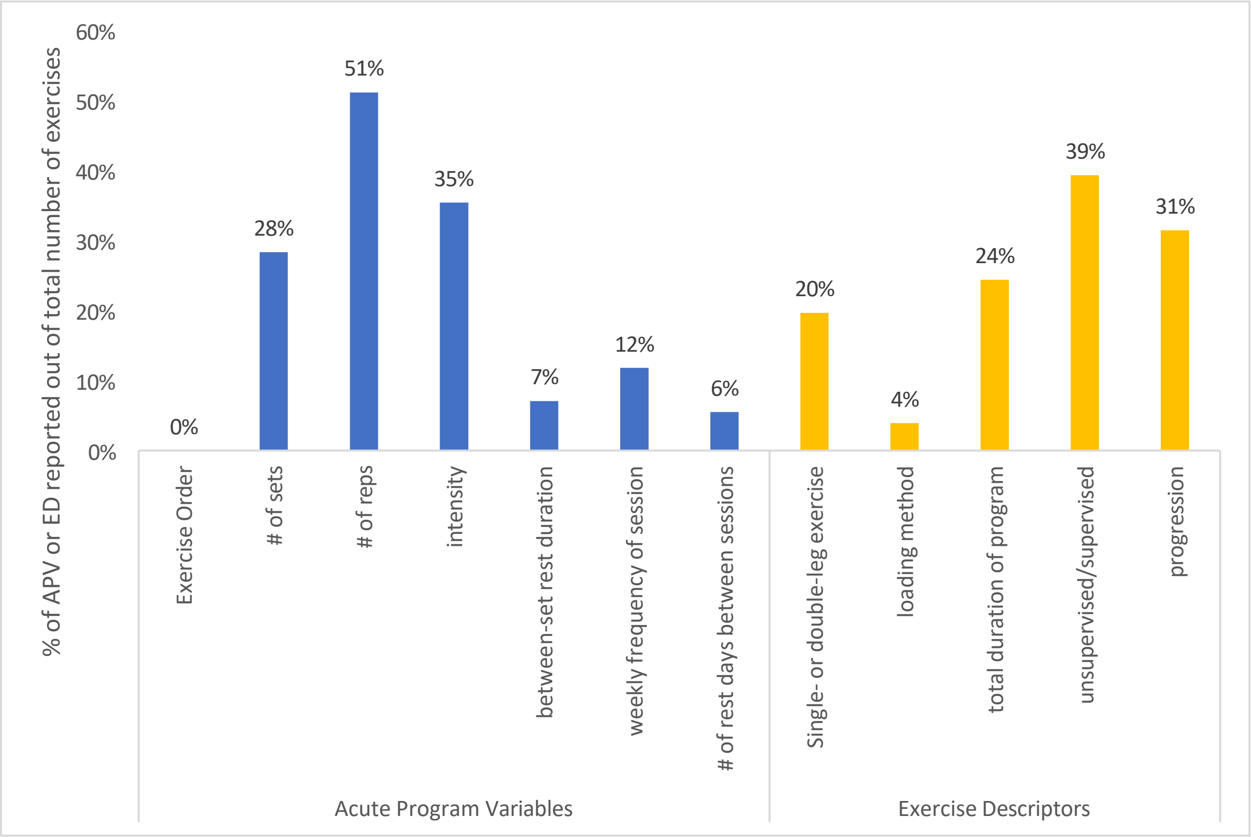

Figure A3: Reporting Frequencies of APVs and EDs

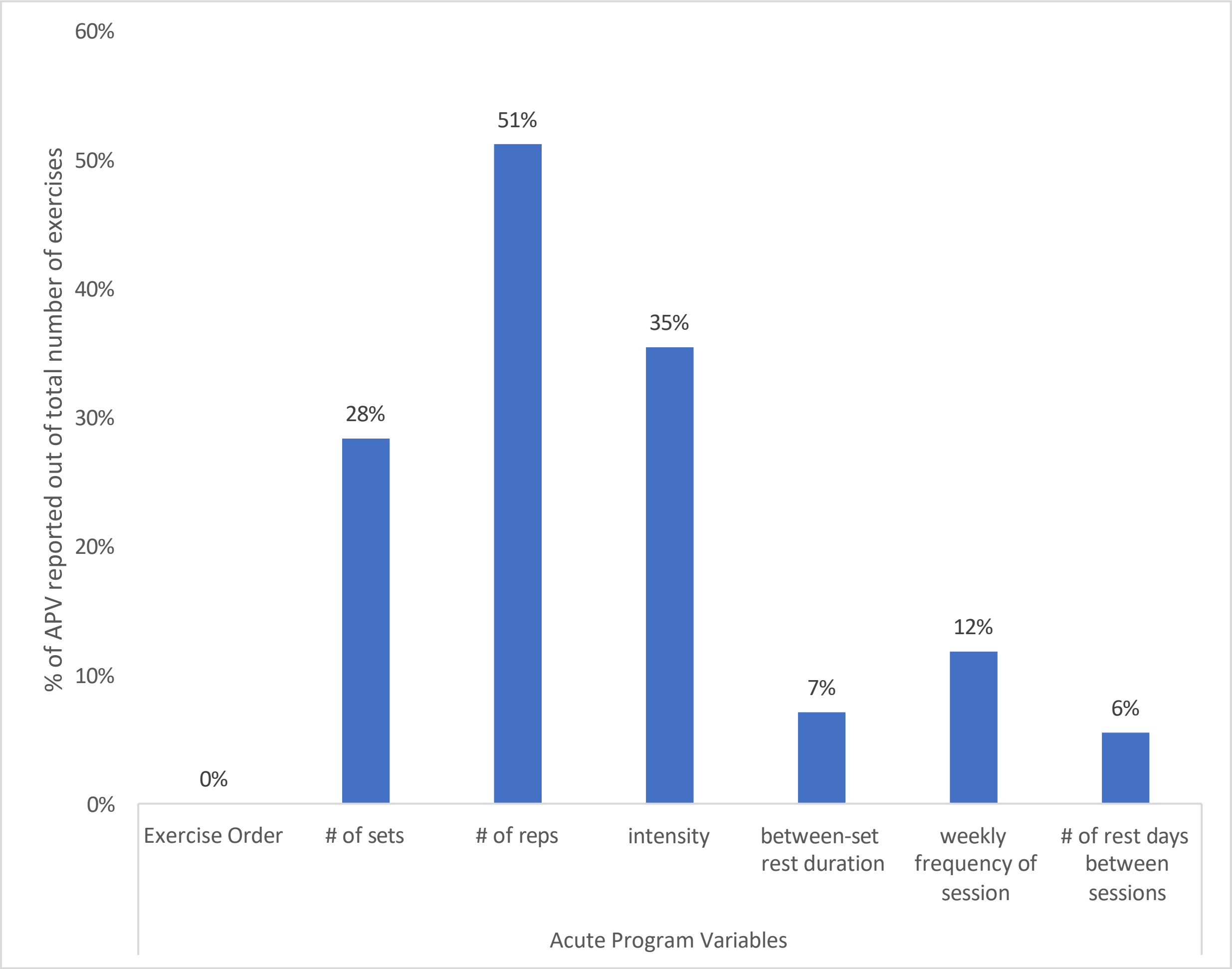

Figure A4: Reporting Frequency of Acute Program Variables. Frequency is calculated by the number of acute program variables reported out of the total possible number of acute program variables that could have been reported across all studies analyzed in this scoping review.

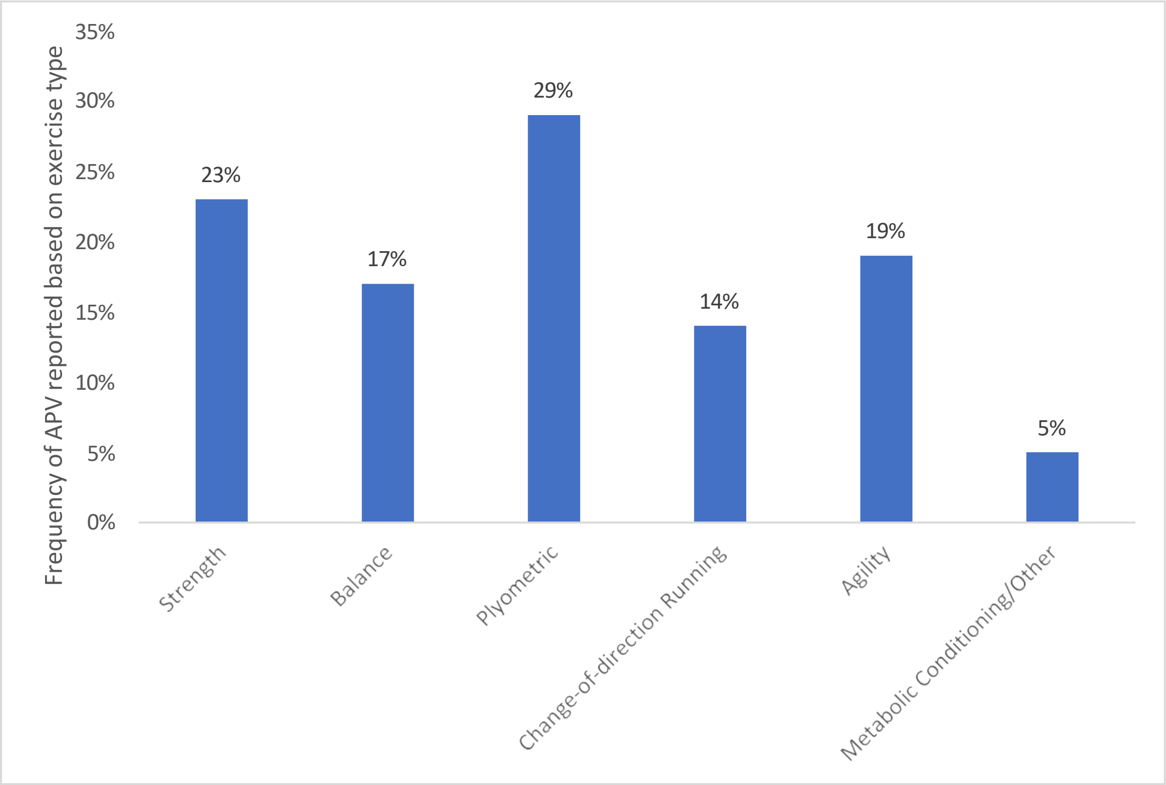

Figure A5: Reporting Frequency of Acute Program Variables by Exercise Type. Frequency is calculated by the number of acute program variables reported out of the total possible number of acute program variables that could have been reported across all studies analyzed in this scoping review for each exercise type.

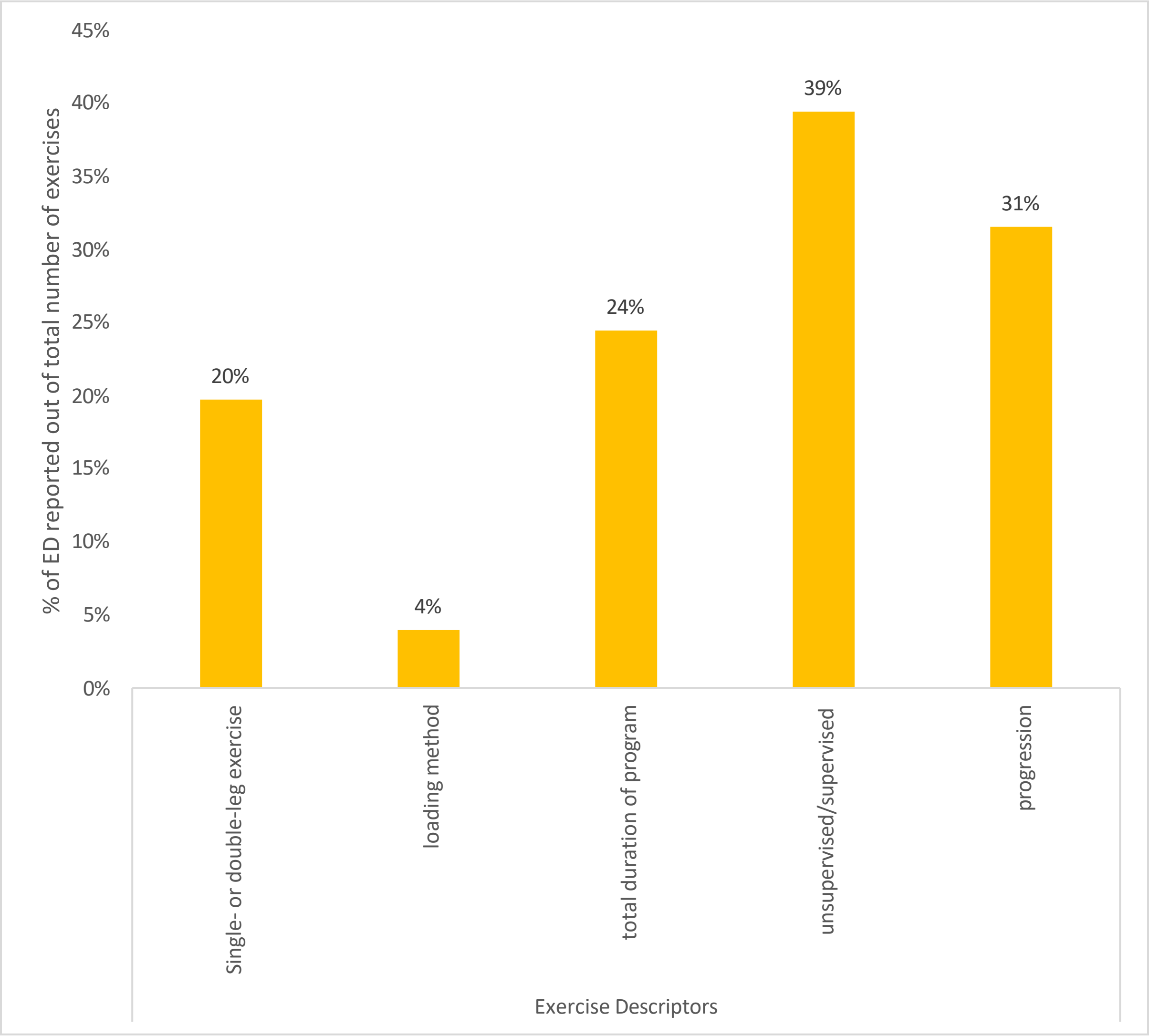

Figure A6: Reporting Frequency of Exercise Descriptors by Exercise Type. Frequency is calculated by the number of exercise descriptors reported out of the total possible number of exercise descriptors that could have been reported across all studies analyzed in this scoping review.

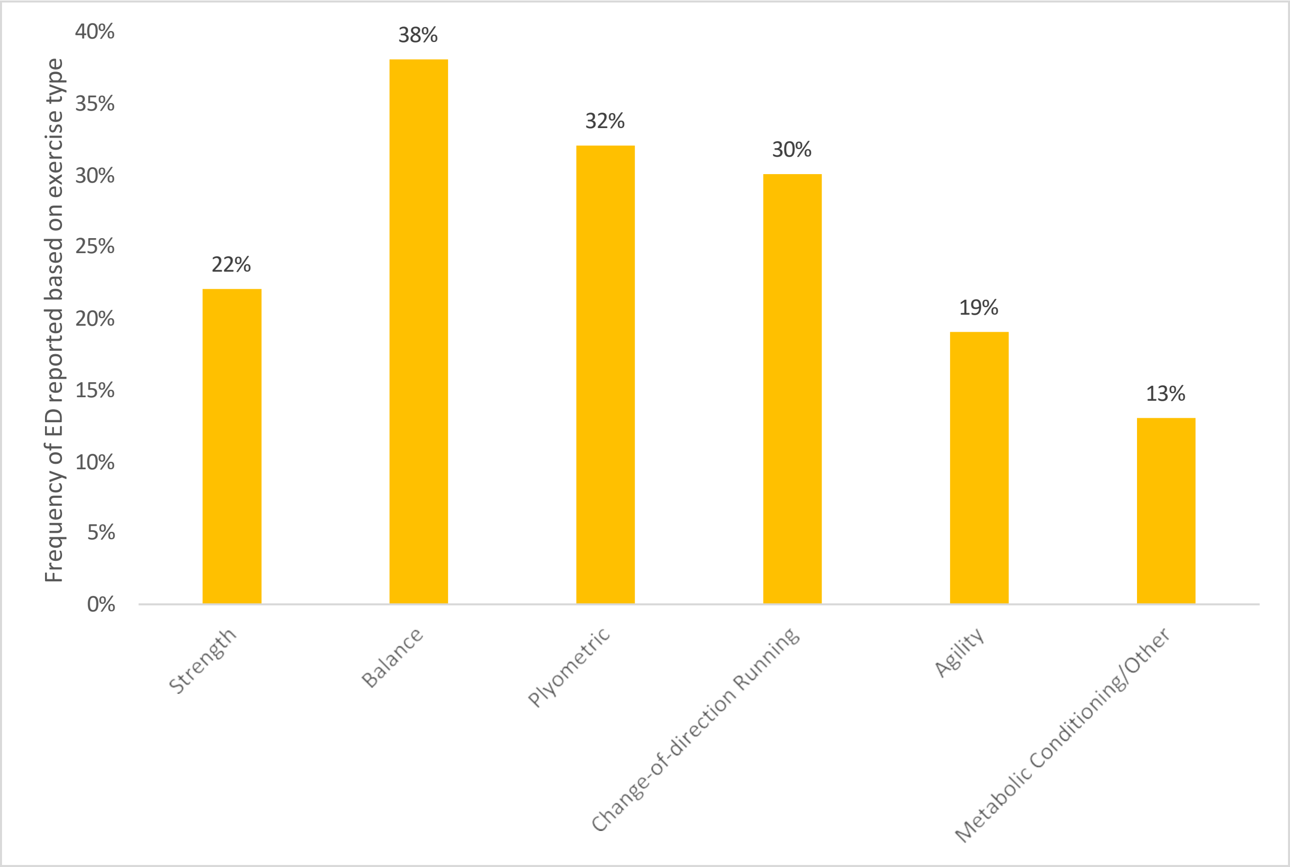

Figure A7: Reporting Frequency of Exercise Descriptors by Exercise Type. Frequency is calculated by the number of exercise descriptors reported out of the total possible number of exercise descriptors that could have been reported across all studies analyzed in this scoping review for each exercise type.

Appendix Table A5: Composition of Rehabilitation Programs

Title	Author	Publication Year	Exercise Type	Exercise	Key Take Away
ACL rehabilitation program using a combined isokinetic and isotonic strengthening protocol	Tsaklis	2002	Strength	11	Accelerated program showed rapid muscle strength restoration without significantly compromising the autograft stability, but did lead to increased synovitis
			Balance	1	
			Plyometric	N/A	
			Change-of-Direction Running	N/A	
			Agility	3	
			Metabolic Conditioning/Other	3	
Rehabilitation after hamstring anterior cruciate ligament reconstruction.	Majima	2002	Strength	5	The combined program of group C appears to have resulted in the best scores among the three programs while indicating that the exclusive use of isokinetic exercises (group B) for strengthening post-ACL knee muscles is not warranted.
			Balance	N/A	
			Plyometric	N/A	
			Change-of-Direction Running	N/A	
			Agility	N/A	
			Metabolic Conditioning/Other	3	
The effects of proprioceptive or strength training on the neuromuscular function of the ACL reconstructed knee: a randomized clinical trial.	Liu-Ambrose	2003	Strength	4	Both protocols influenced Peak Torque Time of hamstring muscles. Load-dependency affected the strength group and sufficient practice influenced the proprioceptive group. Proprioceptive training induces isokinetic gains
			Balance	N/A	
			Plyometric	N/A	
			Change-of-Direction Running	N/A	
			Agility	N/A	
			Metabolic Conditioning/Other	N/A	
Effects of early progressive eccentric exercise on muscle structure after anterior cruciate ligament reconstruction.	Gerber	2007	Strength	5	This study demonstrated that progressive eccentric resistance exercise implemented three weeks after reconstruction of the anterior cruciate ligament can induce changes in the structure of the quadriceps and gluteus maximus that greatly exceed (by more than twofold) those changes following an institutional standard rehabilitation program. These structural increases were observed in both the involved and the uninvolved thighs and with both the semitendinosus-gracilis and the bone-patellar tendon-bone grafts.
			Balance	N/A	
			Plyometric	N/A	
			Change-of-Direction Running	N/A	
			Agility	N/A	
			Metabolic Conditioning/Other	5	
Safety, feasibility, and efficacy of negative work exercise via eccentric muscle activity following anterior cruciate ligament reconstruction.	Gerber	2007	Strength	5	Negative work via eccentric intervention was implemented safely and induced superior short-term results in strength, performance, and activity level post-surgery
			Balance	N/A	
			Plyometric	N/A	
			Change-of-Direction Running	N/A	
			Agility	N/A	
			Metabolic Conditioning/Other	5	
Effects of early progressive eccentric exercise on muscle size and function after anterior cruciate ligament reconstruction: a 1-year follow-up study of a randomized clinical trial.	Gerber	2009	Strength	2	This study demonstrated that the addition of progressive eccentric exercise, implemented 3 weeks after ACL-R, resulted in muscle volume and strength gains in key muscle groups 1 year after surgery that exceeded those changes following a standard rehabilitation program. greater increases in quad and gluteus maximus muscle volume and function in eccentric resistance training compared to standard rehabilitation program after 1 year ACLR
			Balance	N/A	
			Plyometric	N/A	
			Change-of-Direction Running	N/A	
			Agility	N/A	
			Metabolic Conditioning/Other	N/A	
	Souissi	2011	Strength	3	

Improving functional performance and muscle power 4-to-6 months after anterior cruciate li gament reconstruction.			Balance	2	This study introduces a new training modality in rehabilitation after ACLR which results in better recovery of the operated limb along with the contra-lateral leg.
			Plyometric	14	
			Change-of-Direction Running	2	
			Agility	5	
			Metabolic Conditioning/Other	1	
Cross-Exercise on Quadriceps Deficit after ACL Reconstruction	Papandreou	2013	Strength	4	This study demonstrated that CEE used as supplementary to the ACL traditional rehabilitation program in the early stages of reconstruction improves quadriceps muscle strength deficit
			Balance	1	
			Plyometric	1	
			Change-of-Direction Running	N/A	
			Agility	N/A	
			Metabolic Conditioning/Other	4	
The effect of progressive eccentric and concentric training on functional performance after autogenous hamstring anterior cruciate ligament reconstruction: a randomized controlled study	Kinikli	2014	Strength	1	In conclusion, adding a progressive eccentric and concentric exercise program to the standard rehabilitation may improve the functional results after ACL reconstruction with autogenous hamstring grafts.
			Balance	N/A	
			Plyometric	N/A	
			Change-of-Direction Running	N/A	
			Agility	N/A	
			Metabolic Conditioning/Other	N/A	
Report of the Clinical and Functional Primary Outcomes in Men of the ACL-SPORTS Trial: Similar Outcomes in Men Receiving Secondary Prevention With and Without Perturbation Training 1 and 2 Years After ACL Reconstruction	Arundale	2017	Strength	1	The results indicate that perturbation training may not contribute additional benefit to the strengthening, agility, and secondary prevention base of the ACL-SPORTS training program.
			Balance	1	
			Plyometric	N/A	
			Change-of-Direction Running	N/A	
			Agility	N/A	
			Metabolic Conditioning/Other	N/A	
Report of the Primary Outcomes for Gait Mechanics in Men of the ACL-SPORTS Trial: Secondary Prevention With and Without Perturbation Training Does Not Restore Gait Symmetry in Men 1 or 2 Years After ACL Reconstruction	Capin	2017	Strength	N/A	Their findings suggest that a postoperative strength, agility, and secondary prevention training program with or without perturbation training is not effective at ameliorating gait asymmetries in men 1 or 2 years after ACLR. None of the training programs produced significant restoration of interlimb symmetry 1 or 2 years after ACLR.
			Balance	1	
			Plyometric	1	
			Change-of-Direction Running	N/A	
			Agility	1	
			Metabolic Conditioning/Other	N/A	
Differential Effects of 2 Rehabilitation Programs Following Anterior Cruciate Ligament Reconstruction	Setuain	2017	Strength	1	Semitendinosus and Gracilis athropy related to the surgical reconstruction persisted in the reconstructed limb 1 year after medial hamstring ACLR, regardless of whether OCBR or UCR rehabilitation protocols were used. However, the ACBR group showed substantial gains on maximal knee flexor strength and more symmetrical anterior-posterior laxity at the knee joint.
			Balance	N/A	
			Plyometric	N/A	
			Change-of-Direction Running	N/A	
			Agility	1	
			Metabolic Conditioning/Other	N/A	
	Arundale	2018	Strength	2	

Functional and Patient-Reported Outcomes Improve Over the Course of Rehabilitation: A Secondary Analysis of the ACL-SPORTS Trial			Balance	3	This study found that performance of a secondary ACL injury prevention training program during the return-to-sport phase of rehabilitation resulted in significant increases in hop test limb symmetry and PRO scores. The exercises that were common between the groups appeared to be beneficial. Women may need more quadriceps strengthening with a focus on the relationship between QI and risk of reinjury.
			Plyometric	3	
			Change-of-Direction Running	N/A	
			Agility	N/A	
			Metabolic Conditioning/Other	N/A	
Gait Mechanics and Tibiofemoral Loading in Men of the ACL-SPORTS Randomized Control Trial	Capin	2018	Strength	2	Neither SAPP nor SAPP+PERT training appear effective at improving gait mechanics in male athletes in the short term. Meaningful gait asymmetries were mostly resolved after 2 years regardless of the intervention group.
			Balance	1	
			Plyometric	3	
			Change-of-Direction Running	N/A	
			Agility	6	
			Metabolic Conditioning/Other	N/A	
Strength Training Effects on Muscular Regeneration after ACL Reconstruction	Friedmann-Bette	2018	Strength	2	The eccentric overload group had significantly greater muscle hypertrophy than the conventional group and induced a less favorable slower muscle phenotype for strong and fast movements.
			Balance	N/A	
			Plyometric	N/A	
			Change-of-Direction Running	N/A	
			Agility	N/A	
			Metabolic Conditioning/Other	N/A	
Gait Mechanics in Women of the ACL-SPORTS Randomized Control Trial: Interlimb Symmetry Improves over Time Regardless of Treatment Group	Capin	2019	Strength	2	The findings suggest that SAPP training with and without perturbation training does not meaningfully improve walking mechanics among young female athletes. Asymmetrical movement patterns persisted in both groups.
			Balance	1	
			Plyometric	3	
			Change-of-Direction Running	N/A	
			Agility	N/A	
			Metabolic Conditioning/Other	N/A	
Isokinetic eccentric training is more effective than constant load eccentric training for quadriceps rehabilitation following anterior cruciate ligament reconstruction: a randomized controlled trial	Vidmar	2020	Strength	2	Their results support the use of open kinetic chain eccentric training as a safe and effective strategy to the quadriceps muscle rehabilitation after ACL reconstruction. The isometric group showed higher improvements in muscle mass, isometric peak torques, and eccentric peak torques. No differences were found in concentric peak torques, Lysholm scores, or the single leg hop test.
			Balance	N/A	
			Plyometric	N/A	
			Change-of-Direction Running	N/A	
			Agility	N/A	
			Metabolic Conditioning/Other	N/A	
